# Lack of prognostic significance of epidermal growth factor receptor and the oncoprotein p185HER-2 in patients with systemically untreated non-small-cell lung cancer: an immunohistochemical study on cryosections.

**DOI:** 10.1038/bjc.1996.320

**Published:** 1996-07

**Authors:** P. Pfeiffer, P. P. Clausen, K. Andersen, C. Rose

**Affiliations:** Department of Oncology, Odense University Hospital, Denmark.

## Abstract

The prognostic role of the epidermal growth factor receptor (EGFR) and the related receptor p185HER-2 in lung cancer is as yet undefined. We investigated the immunohistochemical expression of EGFR (monoclonal antibody R1; Amersham) and p185HER-2 (polyclonal antibody A485; Dako) in cryosections. A total of 186 unselected and systemically untreated patients with non-small-cell lung cancer (NSCLC) diagnosed and treated at Odense University Hospital, Denmark, were included. Median follow-up period was 66 months. EGFR and p185HER-2 was highly expressed in 55% and 26% of cases respectively. Expression of EGFR was independent of p185HER-2 expression. The expression of EGFR was higher in squamous cell carcinomas whereas the level of p185HER-2 staining was higher in adenocarcinomas. Expression of either or both receptors was not correlated with age, histological grading, stage and prognosis. We conclude that immunohistochemical detection of these growth factor receptors failed to demonstrate a prognostic significance in patients operated on for NSCLC.


British Journal of Cancer (1996) 74, 86-91
© 1996 Stockton Press All rights reserved 0007-0920/96 $12.00

### Lack of prognostic significance of epidermal growth factor receptor and the oncoprotein p185$^{HER-2}$ in patients with systemically untreated non-small-cell lung cancer: an immunohistochemical study on cryosections

P Pfeiffer[1], PP Clausen[2], K Andersen[3] and C Rose[1]

*Departments of [1]Oncology, [2]Pathology and [3]Thoracic Surgery, Odense University Hospital, Denmark.*

**Summary** The prognostic role of the epidermal growth factor receptor (EGFR) and the related receptor p185$^{HER-2}$ in lung cancer is as yet undefined. We investigated the immunohistochemical expression of EGFR (monoclonal antibody R1; Amersham) and p185$^{HER-2}$ (polyclonal antibody A485; Dako) in cryosections. A total of 186 unselected and systemically untreated patients with non-small-cell lung cancer (NSCLC) diagnosed and treated at Odense University Hospital, Denmark, were included. Median follow-up period was 66 months. EGFR and p185$^{HER-2}$ was highly expressed in 55% and 26% of cases respectively. Expression of EGFR was independent of p185$^{HER-2}$ expression. The expression of EGFR was higher in squamous cell carcinomas whereas the level of p185$^{HER-2}$ staining was higher in adenocarcinomas. Expression of either or both receptors was not correlated with age, histological grading, stage and prognosis. We conclude that immunohistochemical detection of these growth factor receptors failed to demonstrate a prognostic significance in patients operated on for NSCLC.

**Keywords:** epidermal growth factor receptor; p185$^{HER-2}$; non-small-cell lung cancer

The improvement in survival for most cancer patients has only been modest and lung cancer is no exception to this rule. Surgical resection alone offers a chance of cure in early-stage non-small-cell lung cancer (NSCLC) (Mountain, 1994). However, some clinical trials indicate possible benefit from adjuvant (Souquet *et al.*, 1993) or neoadjuvant chemotherapy (Rosell *et al.*, 1994; Roth *et al.*, 1994). A prerequisite for further substantiation of these observations is, however, a proper prognostic classification of these patients. A whole cascade of tumour-biological characteristics related to growth, invasion and metastatic potential has been suggested for their possible prognostic value (Gazdar, 1994; Richardson and Johnson, 1993). The main criticisms of most prognostic studies are small sample size, non-homogeneous populations owing to selection bias and use of optimal cut-off values for prognostic variables without a prestated hypothesis (Altman *et al.*, 1994; Simon and Altman, 1994). In addition, various techniques have been employed without proper methodological validation. Not surprisingly, conflicting results have been obtained and it is still not possible to conclude which factors give valid prognostic information.

The protein product of the oncogene *HER*-1, the epidermal growth factor receptor (EGFR), is a 170 kilodalton (kDa) transmembrane protein, exhibiting an extracellular ligand-binding area, a transmembrane domain and an intracellular region with tyrosine kinase activity. The receptor is able to activate cytoplasmic signal proteins that trigger DNA synthesis associated with proliferation and differentiation (Prigent and Lemoine, 1992).

It has recently been shown that the *HER*-2 (or c-*erb*B-2) proto-oncogene encodes a 185 kDa glycoprotein (p185$^{HER-2}$), which has molecular homology with EGFR. Like EGFR, the p185$^{HER-2}$ is a transmembrane receptor with tyrosine kinase activity (Prigent and Lemoine, 1992).

The type 1 (EGFR-related) family of growth factor receptors is important in the regulation of normal cells and in the carcinogenic process (Prigent and Lemoine, 1992), but whether expression of these receptors reflects prognosis remains to be established.

Correspondence: P Pfeiffer, Department of Oncology R, Odense University Hospital, DK-5000 Odense C, Denmark
Received 18 September 1995; revised 5 January 1996; accepted 15 January 1996

To determine whether immunohistochemical detection of EGFR and/or p185$^{HER-2}$ in frozen tissue is of prognostic importance in patients with NSCLC, we conducted the present hypothesis-generating study in a homogeneously treated and unselected cohort of 186 patients. With the exception of two patients, cytotoxic therapy had not been given during the course of the disease.

### Materials and methods

### *Patients and tumour samples*

A total of 186 patients with NSCLC were followed for a median of 66 (40-119) months. The 131 men and 55 women had a median age of 61 (42-79) years at the time of diagnosis. Characteristics of patients were collected from their records (Table I).

All the patients were treated surgically at the Department of Thoracic Surgery at Odense University Hospital, Denmark, from 1984 to 1991. Pulmonary resection was accompanied by intraoperative evaluation of tumour extension with biopsy of suspicious areas and lymph nodes; complete mediastinal lymph node dissection or systematic lymph node sampling was not performed. The stage of the primary tumour (Table I) was determined retrospectively, including a review of the surgical and pathological reports, according to the new International Staging System for lung cancer (Mountain, 1994).

The surgical procedure was considered radical in 152 patients (microscopic radical, 104; macroscopic radical, 48), whereas macroscopic tumour tissue was left in 34 patients. Post-operative adjuvant therapy was not part of the treatment strategy. At the discretion of the treating physician, two patients out of these 186 patients with NSCLC received cytotoxic therapy during the course of their disease. No patient received post-operative adjuvant radiotherapy.

### *Tissue preparation*

Lung tissue was received unfixed in the pathology laboratory immediately after surgical removal. One piece of tumour measuring approximately 1 cm$^3$ was cut out and divided. One part was placed in a cryoconservation tube, snap frozen at

**Table I** Characteristics of 186 patients with NSCLC

| *Parameter* | | n |
|---|---|---|
| Total | | 186 |
| Follow-up (months) | | |
| Median | 66 | |
| Range | 40–119 | |
| Age (years) | | |
| Median | 61 | |
| Range | 42–79 | |
| Male | | 131 |
| Female | | 55 |
| Histological classification | | |
| Squamous cell carcinoma | | 102 |
| Adenocarcinoma | | 59 |
| Large cell carcinoma | | 25 |
| Histological grading | | |
| Highly differentiated | | 15 |
| Moderately differentiated | | 46 |
| Lowly differentiated | | 100 |
| Undifferentiated | | 25 |
| Stage | | |
| I | | 86 |
| II | | 48 |
| IIIa | | 41 |
| IIIb | | 3 |
| IV | | 8 |
| Adjuvant therapy | | |
| Radiotherapy | | 0 |
| Cytotoxic therapy | | 0 |
| Surgical procedure | | |
| Pneumonectomy | | 48 |
| Lobectomy | | 115 |
| Segment resection | | 21 |
| Exploratory thoracotomy | | 2 |
| Radical surgery | | |
| Microscopic | | 104 |
| Macroscopic | | 48 |
| None | | 34 |

−80°C and stored until further examination. The other part was formalin fixed and embedded in paraffin.

*Histological classification*

Haematoxylin–eosin (H&E)-stained sections from formalin-fixed paraffin-embedded tumour specimens were reviewed by one experienced pathologist (PPC). The morphological examination, classification and grading of tumours were performed according to WHO (1981).

*Immunohistochemical analysis*

Before we started immunohistochemical staining of the entire material we performed a series of experiments to select the best antibodies, optimal concentrations and incubation time of reagents. We examined multitissue blocks containing unfixed normal tissue and tumours with a known (low and high) expression of EGFR and p185$^{HER-2}$. Several antibodies produced convincing results but the best signal–noise ratio was obtained with R1 (Amersham, UK) and A485 (Dako, Denmark) respectively.

EGFR and p185$^{HER-2}$ immunostaining was performed on cryostat sections, approximately 5 $\mu$m thick, using the peroxidase-labelled streptavidin–biotin (LSAB) technique. Frozen sections were air dried and fixed in acetone for 10 min (EGFR), or in 4% neutral buffered formaldehyde for 2 min (p185$^{HER-2}$). Tissue sections were then washed twice with Tris-buffered saline (TBS) for 2 min each and incubated with blocking serum [bovine serum albumin (BSA) 2% (Sigma A-7906) in TBS, pH 7.4] for 10 min. Owing to very low background staining, cryostat sections did not require blocking of endogenous peroxidase. All the incubations were performed at room temperature in wet chambers. The blocking serum was drained off and sections were incubated with the primary antibody for 30 min; the optimal dilution had been determined previously (EGFR, 1:100 in 1% BSA/TBS with 15 mM sodium azide, and p185$^{HER-2}$, 1:300 in 1% BSA/TBS with 15 mM sodium azide). For detection of EGFR, we used the murine monoclonal antibody R1. R1 is an $IgG_{2b}$ antibody directed against the extracellular portion of EGFR (Waterfield *et al.*, 1982). p185$^{HER-2}$ was detected by the rabbit polyclonal antibody A485. This antibody was developed against a peptide sequence from the intracytoplasmic part of the human p185$^{HER-2}$.

Sections were washed with TBS and incubated for 30 min with the biotinylated secondary antibodies (anti-mouse E432 and anti-rabbit E433) (also previously titrated for optimal dilutions), followed by streptavidin–horseradish peroxidase (P397) (1:300 in TBS). After washing with TBS the peroxidase activity was visualised by incubation for 20 min in 0.04% 3-amino-9-ethylcarbazole solution containing 0.015% hydrogen peroxide, which gives a red-brown reaction product. After treatment, the sections were washed with distilled water, counterstained with haematoxylin and mounted with Aquamount. Human placenta and normal tonsil tissue were used as positive controls and included in each staining process. Negative control sections of the tumour tissue were immunostained under the same conditions, but omitting the primary antibody; reactions were negative in all cases.

*Immunohistochemical assessment*

After scanning and evaluation of the entire section under low power, a representative area was evaluated with high-power fields (×10 eyepiece, ×40 objective). Scoring of the immunohistochemical results was performed by one author (PP), who was blinded with regard to the clinical data.

The immunohistochemical staining was scored negative if membrane staining was absent. Weak but recognisable staining was classified as grade 1, moderate as grade 2 and strong as grade 3. When there were different intensities within the specimen, the highest grade was recorded. Furthermore, the percentage of positively reacting tumour cells was estimated using a semiquantitative scale ranging from 0% to 100%, with 10 per cent intervals. For further analyses, staining was graded as negative, moderate (<80%) or high (⩾80%). The study was approved by the local ethics committee.

*Statistical evaluation*

Non-parametric statistics were applied. To compare the results of two or more subgroups, the Mann–Whitney (M–W) or Kruskal–Wallis (K–W) tests were used. Correlations between subgroups were assessed by Spearman's rank correlation coefficient ($r_s$) test. Survival curves were generated according to the Kaplan–Meier method and compared by the log-rank test. BMDP statistical software (BMDP/PC Release 7.01, 1993) was used.

### Results

The 186 tumours were distributed as follows: 102 squamous cell carcinoma, 59 adenocarcinoma and 25 large-cell undifferentiated carcinoma (Table I).

*EGFR and p185$^{HER-2}$*

Heterogeneity of tumour staining was present in some biopsies. In addition, in some specimens, a clear difference

**Table II** Relationship between EGFR staining and histological classification in 186 patients with NSCLC

| | No. of patients | EGFR staining None N (%) | Low N (%) | High N (%) |
|---|---|---|---|---|
| SQ | 102 | 6 (6) | 24 (23) | 72 (71) |
| AD | 59 | 7 (12) | 30 (51) | 22 (37) |
| LA | 25 | 1 (4) | 15 (60) | 9 (36) |
| Total | 186 | 14 (8) | 69 (37) | 103 (55) |

SQ, squamous cell carcinoma; AD, adenocarcinoma; LA, large-cell carcinoma. SQ>AD: $P<0.001$. SQ>LA: $P = 0.003$.

**Table III** Relationship between p185$^{HER-2}$ staining and histological classification in 186 patients with NSCLC

| | No. of patients | p185$^{HER-2}$ staining None N (%) | Low N (%) | High N (%) |
|---|---|---|---|---|
| SQ | 102 | 22 (21) | 66 (65) | 14 (14) |
| AD | 59 | 4 (7) | 25 (42) | 30 (51) |
| LA | 25 | 3 (12) | 17 (68) | 5 (20) |
| Total | 186 | 29 (16) | 108 (58) | 49 (26) |

SQ, squamous cell carcinoma; AD, adenocarcinoma; LA, large-cell carcinoma. AD>SQ: $P<0.001$. LA>SQ: $P < 0.001$.

was observed in the immunostaining of EGFR or p185$^{HER-2}$ between central and peripheral tumour cells, the peripheral cells being more often positive. The heterogeneity of immunohistochemical staining was not correlated with the tumour morphology as assessed by routine H&E staining or any other parameter. There was a highly significant correlation between staining intensity and the percentage of positive cells for EGFR as well as p185$^{HER-2}$ (Spearman; $r_s=0.83$, $P<0.00001$). This indicates that both methods may be used in the immunohistochemical estimation of EGFR and p185$^{HER-2}$ content; for the subsequent analyses we have chosen to use percentage of positive tumour cells. There was no association between EGFR and p185$^{HER-2}$ status (Spearman; $r_s=0.004$).

*EGFR* In sections containing bronchial epithelium, we found membrane staining in the basal cells. The majority of tumours stained positively for EGFR; the extent of EGFR expression is shown in Table II. A total of 103 tumours (55%) were strongly positive (staining of ⩾80% of tumour cells), whereas only 14 (8%) were negative.

There was a definite relationship between EGFR status and histology (Table II). Higher expression of EGFR was found in squamous cell carcinomas of the lung than in large cell carcinomas and adenocarcinomas (K−W; $P=0.001$). EGFR content did not show any significant correlation with age, tumour size, lymph node involvement, stage or histological grading.

We found no correlation between EGFR staining and survival in patients with squamous cell carcinoma (relative risk 1.14; 95% I 0.79−1.66), adenocarcinoma of the lung, stage I and/or II, or in the entire group of patients with NSCLC (Figure 1). This was confirmed, whether we applied the cut-off points used in Table II (three groups), used median values (two groups), used quartiles (four groups) or related survival to intensity of staining.

*p185$^{HER-2}$* In the normal bronchial epithelium, membrane staining was most intense at the luminal border. In all, 49 tumours (26%) were strongly positive (staining of ⩾80% of tumour cells), whereas 29 (16%) were negative. p185$^{HER-2}$ status was also related to histology (Table III), but in contrast to EGFR, the level of p185$^{HER-2}$ staining was lower in squamous cell carcinomas than in adenocarcinomas and large cell carcinomas (K−W; $P<0.001$). p185$^{HER-2}$ content was not correlated with age, tumour size, lymph node involvement, stage or histological grading.

**Figure 1** The survival of 186 patients with NSCLC categorised according to EGFR content ($P=0.9$). - - - -, None ($n=14$); ————, low ($n=69$); – – –, high ($n=103$).

**Figure 2** The survival of 186 patients with NSCLC categorised according to p185$^{HER-2}$ content ($P=0.9$). - - - -, None ($n=29$); ————, low ($n=108$); – – –, high ($n=49$).

We found no correlation between p185$^{HER-2}$ staining and survival in patients with squamous cell carcinoma, adenocarcinoma of the lung (relative risk 0.89; 95% CI 0.56−1.41), stage I and/or II, nor in the entire group of patients with NSCLC (Figure 2). This was confirmed, whether we applied the cut-off points for p185$^{HER-2}$ from Table III (three groups), used median values (two groups), used quartiles (four groups) or related survival to intensity of staining.

*In vitro* studies (Kokai *et al.*, 1989) suggested that simultaneous overexpression of both EGFR and p185$^{HER-2}$ acts synergistically. Consequently, we investigated whether patients with EGFR-positive *and* p185$^{HER-2}$-positive tumours might have a poorer prognosis than patients with overexpression of either of the receptors or no overexpression at all. To analyse groups of comparable size we used the median value as cut-off point for both receptors. As for the individual receptor, overexpression of both receptors was of no prognostic significance ($P=0.3$; Figure 3).

### Discussion

A detailed knowledge of prognostic and predictive factors can be essential for predicting patients' outcome and for proper selection of treatment, but also for optimal trial design and for comparison of results. New prognostic factors may help to identify patients with poor prognoses, who might benefit from neoadjuvant (Roth *et al.*, 1994) or post-operative therapy (Holmes, 1993), and patients with good prognoses, who can be spared additional therapy. As new biological markers are constantly being uncovered and investigated, clinicians are faced with the problem: are these markers truly independent prognostic factors? Numerous studies investigating different factors, different cut-off points, different subsets of patients, and different end points are performed, often even without a prespecified hypotheses (Simon and Altman, 1994)

Simon and Altman (1994) have proposed a number of meaningful guidelines that should be fulfilled before definite statements are made concerning the prognostic significance of, for example, the EGF receptor family. In this regard, breast cancers are probably the best studied group of malignant tumours, with several large well-conducted studies which meet most of these guidelines; the majority of large well-conducted studies of breast cancer have found that overexpression of the EGF receptor family is associated with poor prognosis in patients with node-positive breast cancer although results on node-negative patients remain contradictory. This difference may be caused by a treatment effect, i.e. EGFR or p185$^{HER-2}$ are predictive but not prognostic factors (Knoop *et al.*, 1994; Ravdin and Chamness, 1995). By contrast, prognostic studies of the EGF receptor family in NSCLC that include an adequate number of patients are very few.

EGFR and p185$^{HER-2}$ are members of the type 1 (EGFR-related) family of growth factor receptors (Prigent and Lemoine, 1992). These receptors are important in the regulation of normal cell growth, they are connected to the carcinogenic process and they are potential prognostic factors in a variety of human malignant tumours (Gullick, 1991; Prigent and Lemoine, 1992).

**Figure 3** The survival of 186 patients with NSCLC categorised according to EGFR and p185$^{HER-2}$ content ($P=0.38$). - - - -, high EGFR and high p185$^{HER-2}$ ($n=50$); ————, high EGFR or high p185$^{HER-2}$ ($n=103$); – – –, low EGFR and low p185$^{HER-2}$ ($n=33$).

**Table IV** Immunohistochemical detection of EGFR and survival in patients with NSCLC

| *Reference* | *No. of patients* | *SQ* | *AD* | *LA* | *Unknown* | *Prognosis* | *Comments* |
|---|---|---|---|---|---|---|---|
| Dazzi *et al.* (1989) | 152 | 97 | 31 | 7 | 17 | NS | Patients with 'positive tumours' |
| % positive | | 55 | 45 | 43 | 29 | | tended to have improved survival |
| Tateishi *et al.* (1990) | 131 | 131 | | | | NS | |
| % positive | | 42 | | | | | |
| Volm *et al.* (1992) | 81 | 81 | | | | S | Examined 11 potential 'prognostic |
| % positive | | 79 | | | | | factors' |
| Volm *et al.* (1993) | 121 | 121 | | | | S | Stage, EGFR, fos and jun were |
| % positive | | 83 | | | | | prognostic factors |
| Volm and Mattern (1993) | 88 | 88 | | | | S | Evaluated 9 potential 'prognostic |
| % positive | | 77 | | | | | factors' |
| Rusch *et al.* (1993) | 57 | 19 | 31 | 3 | 4 | NS | IH was correlated to mRNA, which |
| % positive | | ? | | | | | had no impact on survival |

IH, immunohistochemistry; S, significant; NS, not significant.

**Table V** Immunohistochemical detection of p185$^{HER-2}$ and survival in patients with NSCLC

| *Reference* | *No. of patients* | *SQ* | *AD* | *LA* | *Unknown* | *Prognosis* | *Comments* |
|---|---|---|---|---|---|---|---|
| Kern *et al.* (1990) | 55 | 16 | 29 | 10 | | S for AD | Significant by multivariate analysis |
| % positive | | 31 | 34 | 0 | | | |
| Tateishi *et al.* (1991) | 203 | 84 | 119 | | | S for AD | |
| % positive | | 2 | 28 | | | | |
| Volm *et al.* (1992) | 81 | 81 | | | | NS | Examined 11 potential 'prognostic |
| % positive | | 36 | | | | | factors' |
| Volm *et al.* (1993) | 121 | 121 | | | | NS | Examined five potential 'prognostic |
| % positive | | 46 | | | | | factors' |
| Volm and Mattern (1993) | 88 | 88 | | | | NS | Examined nine potential 'prognostic |
| % positive | | 35 | | | | | factors' |
| Kern *et al.* (1994) | 46 | | 46 | | | S | Significant by multivariate analysis |
| % positive | | | 34 | | | | |
| Bongiorno *et al.* (1994) | 29 | | 29 | | | NS | |
| % positive | | | 96 | | | | |

IH, immunohistochemistry; S, significant; NS, not significant

We designed this study to test the hypothesis that EGFR and p185$^{HER-2}$ are of prognostic value in patients operated on for NSCLC, and we attempted to meet as many of the above-mentioned guidelines as possible. Furthermore, patients were not treated with adjuvant cytotoxic therapy even, with the exception of 2 patients, when recurrent disease was diagnosed. Thus, we had the possibility of conducting a plain prognostic study.

In agreement with most other studies, we found expression of EGFR in all subtypes of NSCLC, but most frequently in squamous cell carcinomas (Veale *et al.*, 1987; Hendler and Ozanne, 1984; Sobol *et al.*, 1987; Kaseda *et al.*, 1989; Berger *et al.*, 1987) and no correlation between EGFR and the size of the primary tumour, lymph node status or stage (Kaseda *et al.*, 1989; Dittadi *et al.*, 1991; Veale *et al.*, 1989; Di Carlo *et al.*, 1993; Bolufer *et al.*, 1993; Rusch *et al.*, 1993; Dazzi *et al.*, 1989; Volm and Mattern, 1993).

However, when expression of EGFR or p185$^{HER-2}$ has been correlated with outcome, conflicting results are presented. In some studies, overexpression of EGFR has been an indicator of bad prognosis (Volm *et al.*, 1992, 1993; Hendler and Ozanne, 1984; Veale *et al.*, 1987); in some studies expression of EGFR had no impact on outcome (Gorgoulis *et al.*, 1992; Scagliotti *et al.*, 1993; Rusch *et al.*, 1993), whereas still others indicated that patients with EGFR-positive tumours may survive longer than patients without EGFR expression (Dazzi *et al.*, 1989; Veale *et al.*, 1993).

The first immunohistochemical studies to investigate the content of EGFR in patients with NSCLC were based on cryosections (Cerny *et al.*, 1986; Berger *et al.*, 1987; Veale *et al.*, 1987; Sobol *et al.*, 1987). However, almost all subsequent studies used paraffin-embedded tissues. Table IV summarises published immunohistochemical studies that correlate EGFR with outcome in NSCLC. Three studies (Dazzi *et al.*, 1989; Tateishi *et al.*, 1990; Rusch *et al.*, 1993) failed to find a correlation between EGFR expression and prognosis, whereas three other studies, which included patients with squamous cell carcinoma, showed that a high EGFR content was correlated with poor prognosis. However, these three studies, which were all published by Volm *et al.* (Volm *et al.*, 1992, 1993; Volm and Mattern, 1993), do not state how patients were selected, and these different studies might at least partly include the same patients.

The oncogene *HER*-2 and its gene product p185$^{HER-2}$ have also been studied in NSCLC. In accordance with most studies, we found that p185$^{HER-2}$ staining was more prominent in adenocarcinomas than in squamous cell carcinomas and large cell carcinoma (Kern *et al.*, 1990; Tateishi *et al.*, 1991; Shi *et al.*, 1992). In agreement with most studies, we also found no relationship between p185$^{HER-2}$ and the extension of the disease (Volm *et al.*, 1992, 1993; Shi *et al.*, 1992; Bongiorno *et al.*, 1994). Most authors agree that p185$^{HER-2}$ staining is not correlated with prognosis in patients with squamous cell carcinoma (Kern *et al.*, 1990; Volm *et al.*, 1992, 1993; Tateishi *et al.*, 1991). However, a higher content of p185$^{HER-2}$ is correlated with a diagnosis of adenocarcinoma of the lung, and p185$^{HER-2}$ may be a prognostic factor in these adenocarcinomas (Kern *et al.*, 1990, 1994; Tateishi *et al.*, 1991).

Table V summarises published immunohistochemical studies that correlate p185$^{HER-2}$ with survival in NSCLC. Kern *et al.* (1990) examined the expression of p185$^{HER-2}$ in 55 patients with NSCLC and operated on between 1982 and 1985. They found that p185$^{HER-2}$ expression was associated with shortened survival in a subgroup of 29 patients with adenocarcinoma (Kern *et al.*, 1990). In a later study, Kern *et al.* (1994) evaluated the prognostic significance of p185$^{HER-2}$ expression and *ras* gene mutations in 46 patients with pulmonary adenocarcinoma. By univariate and multivariate analysis they showed that p185$^{HER-2}$ expression was associated with shortened survival, whereas K-*ras* mutation approached significance as a poor prognostic indicator. The impact of both p185$^{HER-2}$ expression and a K-*ras* mutation on survival was additive and highly significant. However, these 46 patients with adenocarcinomas were operated on during the same period as the 29 patients previously mentioned (Kern *et al.*, 1990) and this indicates some selection bias. Tateishi *et al.* (1991) examined p185$^{HER-2}$ in 203 patients with NSCLC and found 5-year survival rates of p185$^{HER-2}$-positive and -negative patients of 30% and 52% respectively; they concluded that the expression of p185$^{HER-2}$ may serve as a prognostic indicator in adenocarcinoma of the lung.

Bongiorno *et al.* (1994) used cryosections to evaluate the content of p185$^{HER-2}$ in 29 patients with adenocarcinomas but found no correlation with survival. Except for the study by Bongiorno *et al.* (1994) and ours, all other studies in Table V were based on paraffin-embedded tissue.

It is becoming apparent in breast cancer at least (Ravdin and Chamness, 1995) that overexpression of these receptors is related to response to therapy (i.e. it is a predictive factor), and this may explain, at least to some extent, the conflicting results summarised above.

EGFR and p185$^{HER-2}$ are expressed in NSCLC at a higher level than that of normal bronchial or premalignant tissue (Di Carlo *et al.*, 1993), but without correlation with extension of disease once an invasive tumour has developed. These facts suggest an important step during preinvasive carcinogenesis. It might provide the potential tumour cell with the ability to proliferate when the supply of growth factors is restricted and/or escape terminal differentiation. In addition, if these growth factor receptors and their ligands are important in tumorigenesis, the data suggest that adenocarcinomas, squamous cell carcinomas and large cell carcinoma probably have some carcinogenetic mechanisms in common, regardless of progenitor cell or anatomical location (Weiner *et al.*, 1990).

In conclusion, the present study failed to demonstrate that overexpression of EGFR and p185$^{HER-2}$ is prognostic in systemically untreated patients with NSCLC. Instead EGFR and p185$^{HER-2}$ may be associated with the development of the primary malignant tumour, but without impact on further tumour progression, invasion or development of metastasis. Most studies of EGFR and p185$^{HER-2}$ have used different monoclonal or polyclonal antibody directed toward different epitopes of the receptor and before results are incorporated in the clinical situation, standardisation of the immunohistochemical analyses is therefore indispensable.

### References

ALTMAN DG, LAUSEN B, SAUERBREI W AND SCHUMACHER M. (1994). Dangers of using 'optimal' cutpoints in the evaluation of prognostic factors. *J. Natl Cancer Inst.*, **86**, 829–835.

BERGER MS, GULLICK WJ, GREENFIELD C, EVANS S, ADDIS BJ AND WATERFIELD MD. (1987). Epidermal growth factor receptors in lung tumours. *J. Pathol.*, **152**, 297–307.

BOLUFER P, LLUCH A, MOLINA R, ALBEROLA V, VAZQUEZ C, PADILLA J, GARCIA CONDE J, LLOPIS F AND GUILLEM V. (1993). Epidermal growth factor in human breast cancer, endometrial carcinoma and lung cancer. Its relationship to epidermal growth factor receptor, estradiol receptor and tumor TNM. *Clin. Chim. Acta*, **215**, 51–61.

BONGIORNO PF, WHYTE RI, LESSER EJ, MOORE JH, ORRINGER MB AND BEER DG. (1994). Alterations of K-*ras*, p53, and *erb*B-2/*neu* in human lung adenocarcinomas. *J. Thorac. Cardiovasc. Surg.*, **107**, 590–595.

CERNY T, BARNES DM, HASLETON P, BARBER PV, HEALY K, GULLICK WJ AND THATCHER N. (1986). Expression of epidermal growth factor receptor (EGF-R) in human lung tumours. *Br. J. Cancer*, **54**, 265–269.

DAZZI H, HASLETON PS, THATCHER N, BARNES DM, WILKES S, SWINDELL R AND LAWSON RA. (1989). Expression of epidermal growth factor receptor (EGF-R) in non-small cell lung cancer. Use of archival tissue and correlation of EGF-R with histology, tumour size, node status and survival. *Br. J. Cancer*, **59**, 746–749.

DI CARLO A, MARIANO A, MACCHIA PE, CECERE C, FERRANTE G AND MACCHIA V. (1993). Epidermal growth factor receptor and lipid membrane components in human lung cancers. *J. Endocrinol. Invest.*, **16**, 99–107.

DITTADI R, GION M, PAGAN V, BRAZZALE A, DEL MASCHIO O, BARGOSSI A, BUSETTO A AND BRUSCAGNIN G. (1991). Epidermal growth factor receptor in lung malignancies. Comparison between cancer and normal tissue. *Br. J. Cancer*, **64**, 741–744.

GAZDAR AF. (1994). The molecular and cellular basis of human lung cancer. *Anticancer Res.*, **14**, 261–267.

GORGOULIS V, ANINOS D, MIKOU P, KANAVAROS P, KARAMERIS A, JOAR-DANOGLOU J, RASIDAKIS A, VESLEMES M, OZANNE B AND SPANDIDOS DA. (1992). Expression of EGF, TGF-alpha and EGFR in squamous cell lung carcinomas. *Anticancer Res.*, **12**, 1183–1187.

GULLICK WJ. (1991). Prevalence of aberrant expression of the epidermal growth factor receptor in human cancers. *Br. Med. Bull.*, **47**, 87–98.

HENDLER FJ AND OZANNE B. (1984). Human squamous cell lung cancers express increased epidermal growth factor receptors. *J. Clin. Invest.*, **74**, 647–651.

HOLMES EC. (1993). Postoperative chemotherapy for non-small-cell lung cancer. *Chest*, **103**, 30S–34S.

KASEDA S, UEDA M, OZAWA S, ISHIHARA T, ABE O AND SHIMIZU N. (1989). Expression of epidermal growth factor receptors in four histologic cell types of lung cancer. *J. Surg. Oncol.*, **42**, 16–20.

KERN JA, SCHWARTZ DA, NORDBERG JE, WEINER DB, GREENE MI, TORNEY L AND ROBINSON RA. (1990). p185neu expression in human lung adenocarcinomas predicts shortened survival. *Cancer Res.*, **50**, 5184–5187.

KERN JA, SLEBOS RJ, TOP B, RODENHUIS S, LAGER D, ROBINSON RA, WEINER DB AND SCHWARTZ DA. (1994). C-*erb*B-2 expression and codon 12K-*ras* mutations both predict shortened survival for patients with pulmonary adenocarcinomas. *J. Clin. Invest.*, **93**, 516–520.

KNOOP AS, LAENKHOLM A, MIRZA MR, HANSEN S, THORPE SM AND ROSE C. (1994). Prognostic and predictive factors in early breast cancer. *Proc. ESMO*, **19 (II)**, 9–18.

KOKAI Y, MYERS JN, WADA T, BROWN VI, LEVEA CM, DAVIS JG, DOBASHI K AND GREENE MI. (1989). Synergistic interaction of p185c-neu and the EGF receptor leads to transformation of rodent fibroblasts. *Cell*, **58**, 287–292.

MOUNTAIN CF. (1994). Staging and surgical treatment of lung cancer. *Adv. Oncol.*, **9**, 10–14.

PRIGENT SA AND LEMOINE NR. (1992). The type 1 (EGFR-related) family of growth factor receptors and their ligands. *Prog. Growth Factor Res.*, **4**, 1–24.

RAVDIN PM AND CHAMNESS GC. (1995). The c-*erb*B-2 proto-oncogene as a prognostic and predictive marker in breast cancer: a paradigm for the development of other macromolecular markers – a review. *Gene*, **159**, 19–27.

RICHARDSON GE AND JOHNSON BE. (1993). The biology of lung cancer. *Semin. Oncol.*, **20**, 105–127.

ROSELL R, GOMEZ CODINA J, CAMPS C, MAESTRE J, PADILLE J, CANTO A, MATE JL, LI S, ROIG J, OLAZABAL A, CANELA M, ARIZA A, SKACEL Z, MORERA-PRAT J AND ABAD A. (1994). A randomized trial comparing preoperative chemotherapy plus surgery with surgery alone in patients with non-small-cell lung cancer [see comments]. *N. Engl. J. Med.*, **330** 153–158.

ROTH JA, FOSSELLA F, KOMAKI R, RYAN MB, PUTNAM JB JR, LEE JS, DHINGRA H, DE CARO L, CHASEN M, MCGAVRAN M, ATKINSON EN AND HONG WK. (1994). A randomized trial comparing perioperative chemotherapy and surgery with surgery alone in resectable stage IIIA non-small-cell lung cancer. *J. Natl Cancer Inst.*, **86**, 673–680.

RUSCH V, BASELGA J, CORDON-CARDO C, ORAZEM J, ZAMAN M, HODA S, MCINTOSH J, KURIE J AND DMITROVSKY E. (1993). Differential expression of the epidermal growth factor receptor and its ligands in primary non-small cell lung cancers and adjacent benign lung. *Cancer Res.*, **53**, 2379–2385.

SCAGLIOTTI GV, LEONARDO E, CAPPIA S, MASIERO P, MICELA M, GUBETTA L AND POZZI E. (1993). Epidermal growth factor receptor and *neu*-oncogene expression in lung cancer. *Proc. ASCO*, **12**, 328.

SHI D, HE G, CAO S, PAN W, ZHANG HZ, YU D AND HUNG MC. (1992). Overexpression of the c-erbB-2/neu-encoded p185 protein in primary lung cancer. *Mol. Carcinogen.*, **5**, 213–218.

SIMON R AND ALTMAN DG. (1994). Statistical aspects of prognostic factor studies in oncology [editorial]. *Br. J. Cancer*, **69**, 979–985.

SOBOL RE, ASTARITA RW, HOFEDITZ C, MASUI H, FAIRSHTER R, ROYSTON I AND MENDELSOHN J. (1987). Epidermal growth factor receptor expression in human lung carcinomas defined by a monoclonal antibody. *J. Natl Cancer Inst.*, **79**, 403–405.

SOUQUET PJ, CHAUVIN F, BOISSEL JP, CELLERINO R, CORMIER Y, GANZ PA, KAASA S, PATER JL, QUOIX E, RAPP E, TUMARELLO D, WILLIAMS J, WOODS BL AND BERNARD JP. (1993). Polychemotherapy in advanced non small cell lung cancer: a meta-analysis. *Lancet*, **342**, 19–21.

TATEISHI M, ISHIDA T, MITSUDOMI T, KANEKO S AND SUGIMACHI K. (1990). Immunohistochemical evidence of autocrine growth factors in adenocarcinoma of the human lung. *Cancer Res.*, **50**, 7077–7080.

TATEISHI M, ISHIDA T, MITSUDOMI T, KANEKO S AND SUGIMACHI K. (1991). Prognostic value of c-erbB-2 protein expression in human lung adenocarcinoma and squamous cell carcinoma. *Eur. J. Cancer*, **27**, 1372–1375.

VEALE D, ASHCROFT T, MARSH C, GIBSON GJ AND HARRIS AL. (1987). Epidermal growth factor receptors in non-small cell lung cancer. *Br. J. Cancer*, **55**, 513–516.

VEALE D, KERR N, GIBSON GJ AND HARRIS AL. (1989). Characterization of epidermal growth factor receptor in primary human non-small cell lung cancer. *Cancer Res.*, **49**, 1313–1317.

VEALE D, KERR N, GIBSON GJ, KELLY PJ AND HARRIS AL. (1993). The relationship of quantitative epidermal growth factor receptor in non-small cell lung cancer to long term survival. *Br. J. Cancer*, **68**, 162–165.

VOLM M, EFFERTH T AND MATTERN J. (1992). Oncoprotein (c-myc, c-erbB1, c-erbB2, c-fos) and suppressor gene product (p53) expression in squamous cell carcinomas of the lung. Clinical and biological correlations. *Anticancer Res.*, **12**, 11–20.

VOLM M AND MATTERN J. (1993). Correlation between successful heterotransplantation of lung tumors in nude mice, poor prognosis of patients and expression of Fos, Jun, ErbB1, and Ras. *Anticancer Res.*, **13**, 2021–2025.

VOLM M, DRINGS P AND WODRICH W. (1993). Prognostic significance of the expression of c-*fos*, c-*jun* and c-*erb*B-1 oncogene products in human squamous cell lung carcinomas. *J. Cancer Res. Clin. Oncol.*, **119**, 507–510.

WATERFIELD MD, MAYES EL, STROOBANT P, BENNET PL, YOUNG S, GOODFELLOW PN, BANTING GS AND OZANNE B. (1982). A monoclonal antibody to the human epidermal growth factor receptor. *J. Cell. Biochem.*, **20**, 149–161.

WEINER DB, NORDBERG J, ROBINSON R, NOWELL PC, GAZDAR A, GREENE MI, WILLIAMS WV, COHEN JA AND KERN JA. (1990). Expression of the *neu* gene-encoded protein (P185neu) in human non-small cell carcinomas of the lung. *Cancer Res.*, **50**, 421–425.

WORLD HEALTH ORGANIZATION. (1981). Histological typing of lung tumours. *Tumori*, **67**, 253–272.


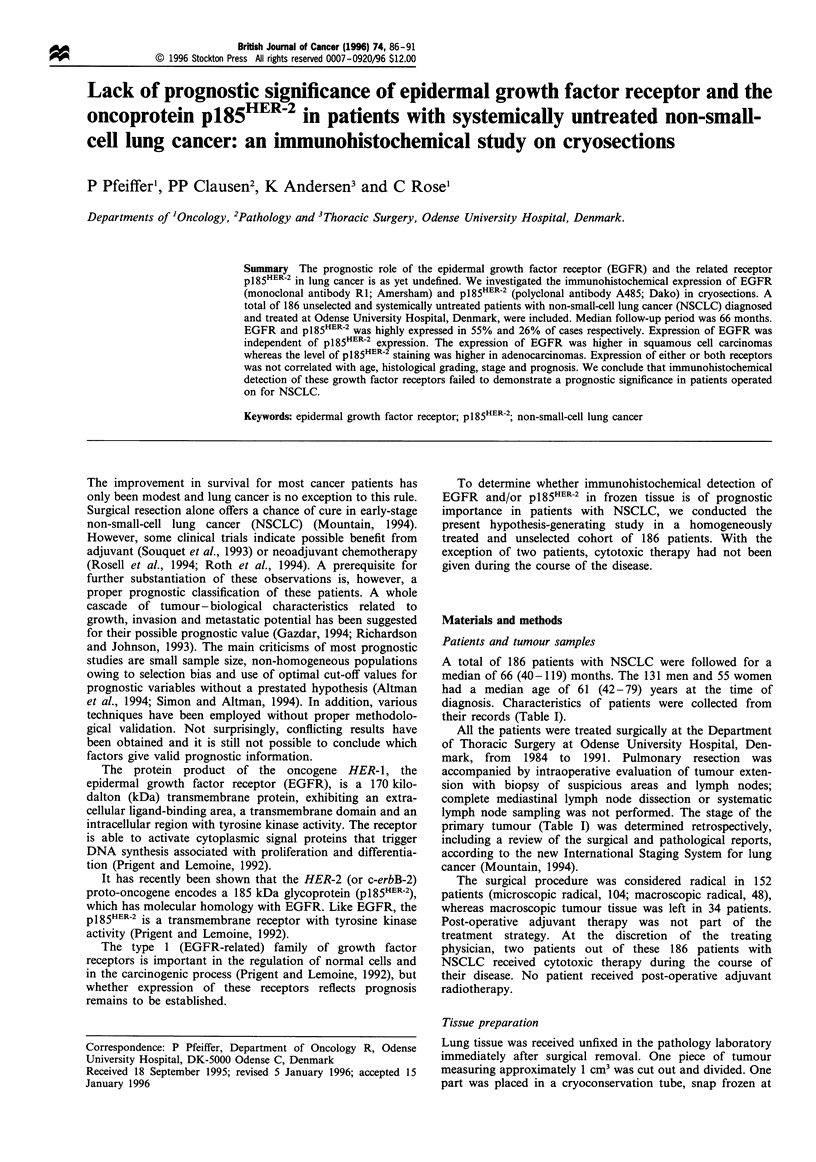

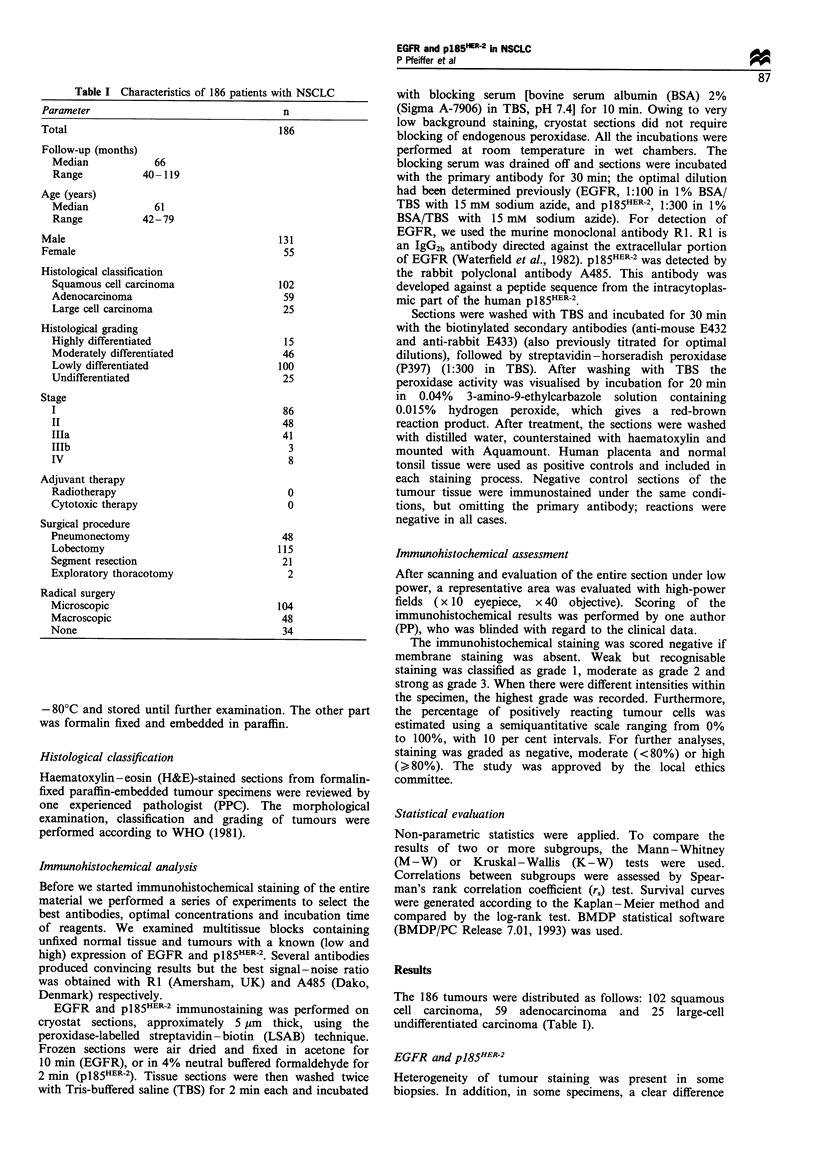

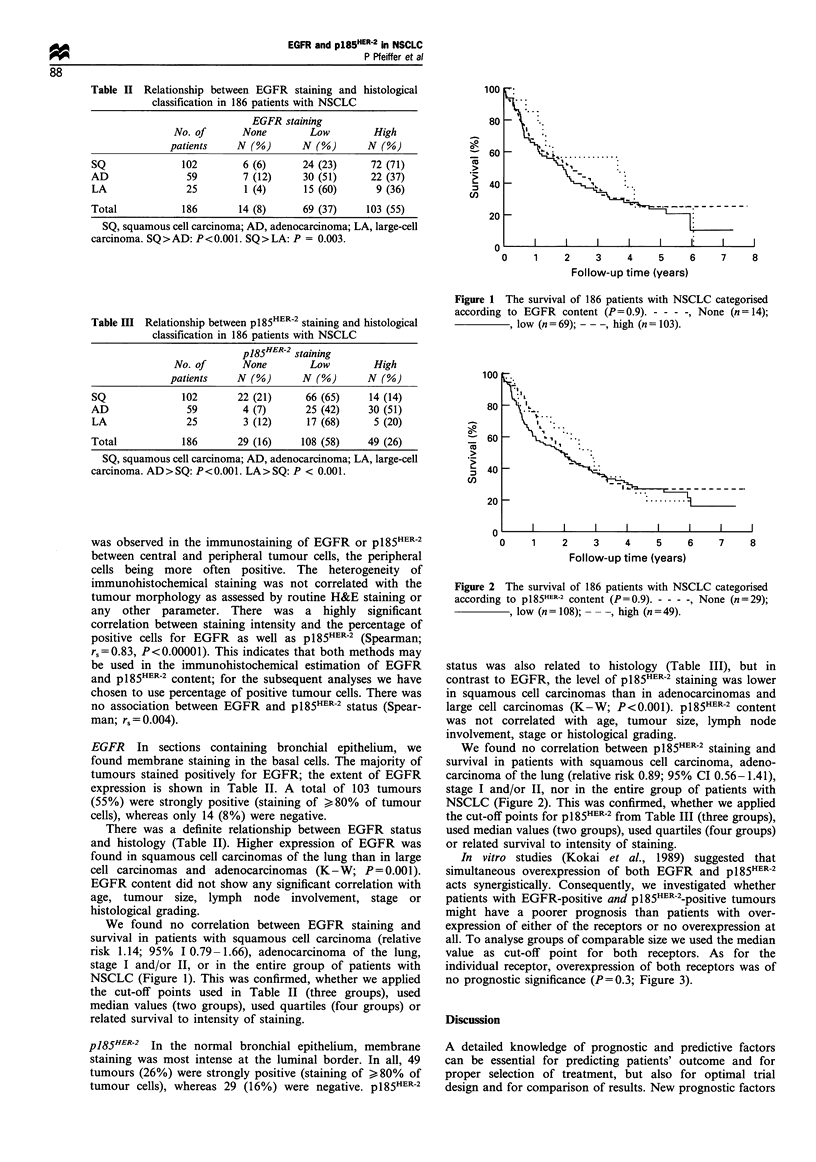

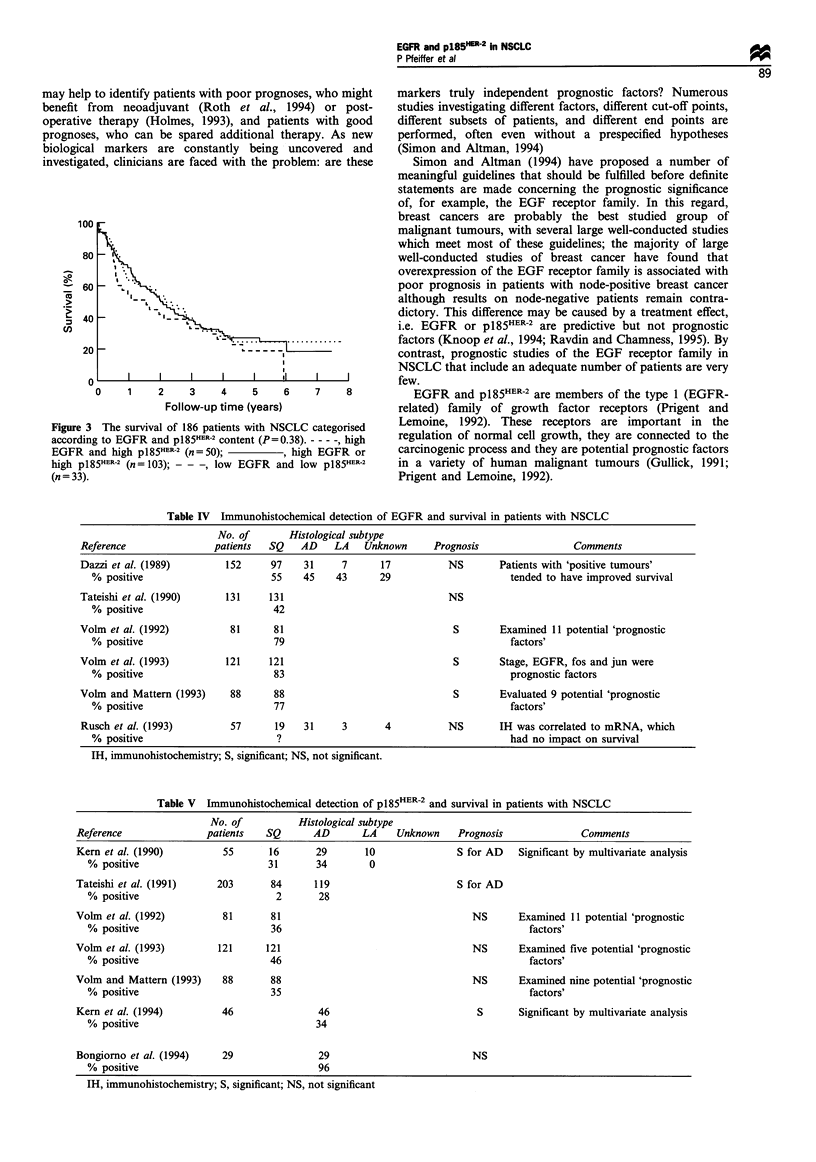

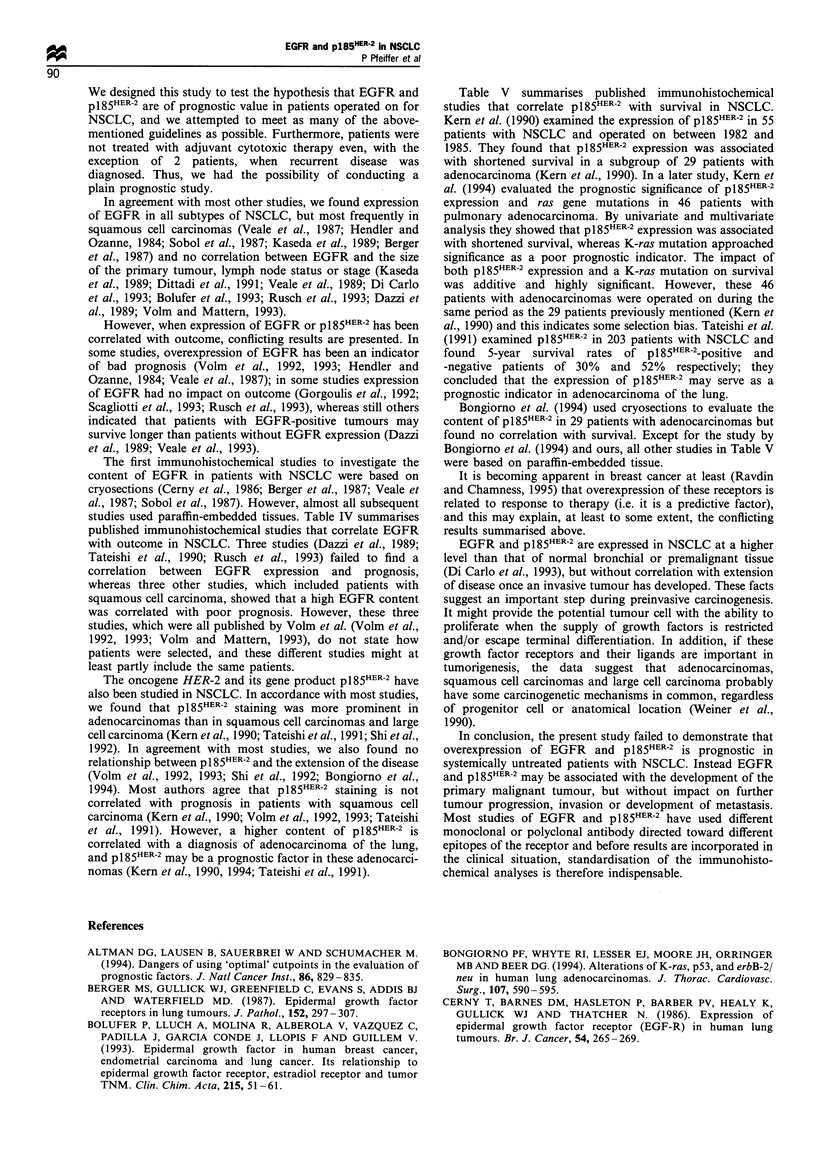

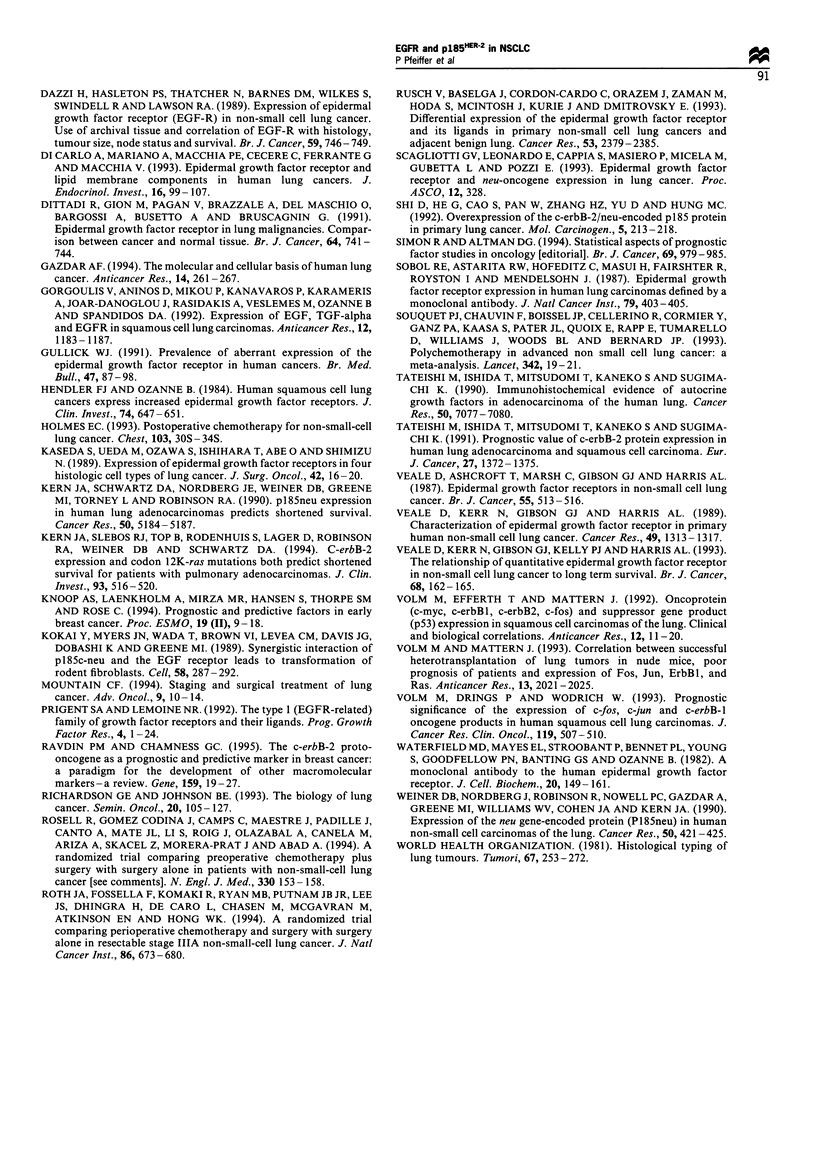


## References

[OCR_00792] Altman D. G., Lausen B., Sauerbrei W., Schumacher M. (1994). Dangers of using "optimal" cutpoints in the evaluation of prognostic factors.. J Natl Cancer Inst.

[OCR_00798] Berger M. S., Gullick W. J., Greenfield C., Evans S., Addis B. J., Waterfield M. D. (1987). Epidermal growth factor receptors in lung tumours.. J Pathol.

[OCR_00800] Bolufer P., Lluch A., Molina R., Alberola V., Vazquez C., Padilla J., Garcia-Conde J., Llopis F., Guillem V. (1993). Epidermal growth factor in human breast cancer, endometrial carcinoma and lung cancer. Its relationship to epidermal growth factor receptor, estradiol receptor and tumor TNM.. Clin Chim Acta.

[OCR_00811] Bongiorno P. F., Whyte R. I., Lesser E. J., Moore J. H., Orringer M. B., Beer D. G. (1994). Alterations of K-ras, p53, and erbB-2/neu in human lung adenocarcinomas.. J Thorac Cardiovasc Surg.

[OCR_00818] Cerny T., Barnes D. M., Hasleton P., Barber P. V., Healy K., Gullick W., Thatcher N. (1986). Expression of epidermal growth factor receptor (EGF-R) in human lung tumours.. Br J Cancer.

[OCR_00828] Dazzi H., Hasleton P. S., Thatcher N., Barnes D. M., Wilkes S., Swindell R., Lawson R. A. (1989). Expression of epidermal growth factor receptor (EGF-R) in non-small cell lung cancer. Use of archival tissue and correlation of EGF-R with histology, tumour size, node status and survival.. Br J Cancer.

[OCR_00834] Di Carlo A., Mariano A., Macchia P. E., Cecere C., Ferrante G., Macchia V. (1993). Epidermal growth factor receptor and lipid membrane components in human lung cancers.. J Endocrinol Invest.

[OCR_00839] Dittadi R., Gion M., Pagan V., Brazzale A., Del Maschio O., Bargossi A., Busetto A., Bruscagnin G. (1991). Epidermal growth factor receptor in lung malignancies. Comparison between cancer and normal tissue.. Br J Cancer.

[OCR_00844] Gazdar A. F. (1994). The molecular and cellular basis of human lung cancer.. Anticancer Res.

[OCR_00851] Gorgoulis V., Aninos D., Mikou P., Kanavaros P., Karameris A., Joardanoglou J., Rasidakis A., Veslemes M., Ozanne B., Spandidos D. A. (1992). Expression of EGF, TGF-alpha and EGFR in squamous cell lung carcinomas.. Anticancer Res.

[OCR_00855] Gullick W. J. (1991). Prevalence of aberrant expression of the epidermal growth factor receptor in human cancers.. Br Med Bull.

[OCR_00860] Hendler F. J., Ozanne B. W. (1984). Human squamous cell lung cancers express increased epidermal growth factor receptors.. J Clin Invest.

[OCR_00867] Holmes E. C. (1993). Postoperative chemotherapy for non-small-cell lung cancer.. Chest.

[OCR_00871] Kaseda S., Ueda M., Ozawa S., Ishihara T., Abe O., Shimizu N. (1989). Expression of epidermal growth factor receptors in four histologic cell types of lung cancer.. J Surg Oncol.

[OCR_00876] Kern J. A., Schwartz D. A., Nordberg J. E., Weiner D. B., Greene M. I., Torney L., Robinson R. A. (1990). p185neu expression in human lung adenocarcinomas predicts shortened survival.. Cancer Res.

[OCR_00882] Kern J. A., Slebos R. J., Top B., Rodenhuis S., Lager D., Robinson R. A., Weiner D., Schwartz D. A. (1994). C-erbB-2 expression and codon 12 K-ras mutations both predict shortened survival for patients with pulmonary adenocarcinomas.. J Clin Invest.

[OCR_00895] Kokai Y., Myers J. N., Wada T., Brown V. I., LeVea C. M., Davis J. G., Dobashi K., Greene M. I. (1989). Synergistic interaction of p185c-neu and the EGF receptor leads to transformation of rodent fibroblasts.. Cell.

[OCR_00904] Prigent S. A., Lemoine N. R. (1992). The type 1 (EGFR-related) family of growth factor receptors and their ligands.. Prog Growth Factor Res.

[OCR_00909] Ravdin P. M., Chamness G. C. (1995). The c-erbB-2 proto-oncogene as a prognostic and predictive marker in breast cancer: a paradigm for the development of other macromolecular markers--a review.. Gene.

[OCR_00915] Richardson G. E., Johnson B. E. (1993). The biology of lung cancer.. Semin Oncol.

[OCR_00917] Rosell R., Gómez-Codina J., Camps C., Maestre J., Padille J., Cantó A., Mate J. L., Li S., Roig J., Olazábal A. (1994). A randomized trial comparing preoperative chemotherapy plus surgery with surgery alone in patients with non-small-cell lung cancer.. N Engl J Med.

[OCR_00928] Roth J. A., Fossella F., Komaki R., Ryan M. B., Putnam J. B., Lee J. S., Dhingra H., De Caro L., Chasen M., McGavran M. (1994). A randomized trial comparing perioperative chemotherapy and surgery with surgery alone in resectable stage IIIA non-small-cell lung cancer.. J Natl Cancer Inst.

[OCR_00935] Rusch V., Baselga J., Cordon-Cardo C., Orazem J., Zaman M., Hoda S., McIntosh J., Kurie J., Dmitrovsky E. (1993). Differential expression of the epidermal growth factor receptor and its ligands in primary non-small cell lung cancers and adjacent benign lung.. Cancer Res.

[OCR_00946] Shi D., He G., Cao S., Pan W., Zhang H. Z., Yu D., Hung M. C. (1992). Overexpression of the c-erbB-2/neu-encoded p185 protein in primary lung cancer.. Mol Carcinog.

[OCR_00951] Simon R., Altman D. G. (1994). Statistical aspects of prognostic factor studies in oncology.. Br J Cancer.

[OCR_00957] Sobol R. E., Astarita R. W., Hofeditz C., Masui H., Fairshter R., Royston I., Mendelsohn J. (1987). Epidermal growth factor receptor expression in human lung carcinomas defined by a monoclonal antibody.. J Natl Cancer Inst.

[OCR_00963] Souquet P. J., Chauvin F., Boissel J. P., Cellerino R., Cormier Y., Ganz P. A., Kaasa S., Pater J. L., Quoix E., Rapp E. (1993). Polychemotherapy in advanced non small cell lung cancer: a meta-analysis.. Lancet.

[OCR_00969] Tateishi M., Ishida T., Mitsudomi T., Kaneko S., Sugimachi K. (1990). Immunohistochemical evidence of autocrine growth factors in adenocarcinoma of the human lung.. Cancer Res.

[OCR_00973] Tateishi M., Ishida T., Mitsudomi T., Kaneko S., Sugimachi K. (1991). Prognostic value of c-erbB-2 protein expression in human lung adenocarcinoma and squamous cell carcinoma.. Eur J Cancer.

[OCR_00979] Veale D., Ashcroft T., Marsh C., Gibson G. J., Harris A. L. (1987). Epidermal growth factor receptors in non-small cell lung cancer.. Br J Cancer.

[OCR_00986] Veale D., Kerr N., Gibson G. J., Harris A. L. (1989). Characterization of epidermal growth factor receptor in primary human non-small cell lung cancer.. Cancer Res.

[OCR_00991] Veale D., Kerr N., Gibson G. J., Kelly P. J., Harris A. L. (1993). The relationship of quantitative epidermal growth factor receptor expression in non-small cell lung cancer to long term survival.. Br J Cancer.

[OCR_01009] Volm M., Drings P., Wodrich W. (1993). Prognostic significance of the expression of c-fos, c-jun and c-erbB-1 oncogene products in human squamous cell lung carcinomas.. J Cancer Res Clin Oncol.

[OCR_00997] Volm M., Efferth T., Mattern J. (1992). Oncoprotein (c-myc, c-erbB1, c-erbB2, c-fos) and suppressor gene product (p53) expression in squamous cell carcinomas of the lung. Clinical and biological correlations.. Anticancer Res.

[OCR_01003] Volm M., Mattern J. (1993). Correlation between successful heterotransplantation of lung tumors in nude mice, poor prognosis of patients and expression of Fos, Jun, ErbB1, and Ras.. Anticancer Res.

[OCR_01013] Waterfield M. D., Mayes E. L., Stroobant P., Bennet P. L., Young S., Goodfellow P. N., Banting G. S., Ozanne B. (1982). A monoclonal antibody to the human epidermal growth factor receptor.. J Cell Biochem.

[OCR_01019] Weiner D. B., Nordberg J., Robinson R., Nowell P. C., Gazdar A., Greene M. I., Williams W. V., Cohen J. A., Kern J. A. (1990). Expression of the neu gene-encoded protein (P185neu) in human non-small cell carcinomas of the lung.. Cancer Res.

